# Advanced Trauma Life Support Course Delivery: Comparison of Outcomes From Modifications During Covid-19

**DOI:** 10.7759/cureus.16811

**Published:** 2021-08-01

**Authors:** Lauren Dyer, Luis Llerena, Michael Brannick, John R Lunde, Frank Whitaker

**Affiliations:** 1 College of Arts and Sciences, University of South Florida, Tampa, USA; 2 Department of Surgery, University of South Florida, Tampa, USA; 3 Center for Advanced Medical Learning and Simulation, University of South Florida Morsani College of Medicine, Tampa, USA; 4 Division of Critical Care Medicine, Orange Park Medical Center, Jacksonville, USA

**Keywords:** advanced trauma life support, medical education, hybrid course, matls, medical simulation

## Abstract

Introduction

The Advanced Trauma Life Support (ATLS) Course is a two-day long medical training course developed by the American College of Surgeons (ACS) to help train and prepare healthcare providers to care for severely injured patients. The coronavirus disease 2019 (COVID-19) pandemic has resulted in the modification or cancellation of many education programs across the world. At the University of South Florida’s Center for Advanced Medical Learning and Simulation (CAMLS) two different models of ATLS were delivered in response to the COVID-19 pandemic with both models utilizing the ACS’s online mobile ATLS (mATLS). In this study three different models of ATLS delivered by USF CAMLS between 2019 and 2020 were compared to determine if there were any impacts on the education and functionality of the ATLS course between the three different models of ATLS: a baseline ATLS course, an augmented ATLS course that used mATLS, and an ATLS course that used mATLS as a replacement for in-person lectures.

Material and methods

To compare the three models of ATLS delivery, a total of six ATLS courses were studied: a baseline face-to-face ATLS course from June 2019, two Mobile ATLS (mATLS) courses from September 2020, and three augmented ATLS courses that contained both face-to-face and mATLS delivery from October, November and December 2020. The only differences between the traditional ATLS courses from 2020 and the pre-COVID ATLS course from 2019 were that the courses from 2020 utilized mATLS and that the course days were longer due to cleaning time. These courses were selected to have a non-significant difference in the number of learners in each model of ATLS course. The data that were collected from these courses included: post-test results from learners, learner feedback surveys, and interviews with the ATLS Course Director, ATLS Course Coordinator, and the Educational Coordinator.

Results

The only courses with significant differences in the post-test mean scores were for the baseline ATLS course compared to the mATLS courses. The augmented courses showed similar post-test performance to the mATLS courses. Students viewed the courses favorably with the only major complaint between the 2019 and the 2020 courses being a high amount of downtime for the 2020 courses due to time required to disinfect skill stations and equipment. The main difficulties for the ATLS Course Director, ATLS Course Coordinator, and the Educational Coordinator with the ATLS courses in 2020 were concerned with challenges from COVID-19, like social distancing, and not with mATLS or the shortened instruction time with the hybrid model.

Discussion

This preliminary study analyzed three delivery models of ATLS. The mATLS may be able to replace in-person lectures of ATLS courses as courses using alternative delivery formats showed post-test scores as good or better than the baseline face-to-face course.

## Introduction

The outbreak of coronavirus disease 2019 (COVID-19) has impacted various areas of life with the implementation on social distancing guidelines, and medical education has not been excluded. As a result of the COVID-19 pandemic, the traditional face-to-face methods for the delivery of medical training had to be reevaluated and modified in order to align with the recommendations of public health officials [1-3].

Before the outbreak of the novel coronavirus the Advanced Trauma Life Support (ATLS) course, which is a course developed by the American College of Surgeon (ACS) Committee on Trauma (COT), was delivered entirely in a face-to-face environment over the course of two days. The two days of the ATLS would contain didactic lectures, skill stations, a multiple-choice question post-test, and a skills assessment that the learners must pass in order to become certified ATLS providers.

Mobile ATLS (mATLS) was released by the ACS COT in 2018 to aid training sites in educating ATLS learners. During the outbreak of COVID-19, mATLS gained relevance as a way to continue ATLS instruction. mATLS is an online program containing 13 modules with these modules covering the same information traditionally discussed in the ATLS didactic lectures [4].

This study evaluated the effectiveness of mATLS in training Post Graduate Year One (PGY-1) surgical and emergency medicine interns by comparing the test results and evaluations of three delivery models of the ATLS: pre-COVID baseline course, augmented course with the addition of mATLS, and d course with mATLS.

Literature review

There has been an increasing trend for the utilization of digital learning and the flipped classroom model in medical and postgraduate medical education [1,2,5,6]. The COVID-19 pandemic has increased the need for innovative methods to train students while maintaining the health and safety guidance from public health officials.

The usage of digital learning for ATLS has been researched in various aspects. For example, the usage of telemedicine would allow physicians from rural areas to participate in the ATLS course without having to travel to a teaching site [7]. No significant differences were found between the face-to-face and telemedicine ATLS groups in regards to the post-test results, evaluations of student performance at the skill stations, or course pass rates and participant feedback [7]. In addition to a digital medium being used for skill station instruction, the post-test for the ATLS course has been evaluated [8]. An online post-test was found to be feasible as a method of administrating the ATLS post-test and, compared to the traditional paper and pencil administration of the exam, the online exam was able to better allow for psychometric analysis of the post-test [9].

Several proposals have been made in order to counteract the decreased in-person activities yet still maintain the education benchmarks and requirements for students and residents during the pandemic [1,2]. Tools such as virtual and augmented reality as well as telemedicine have been proposed in order to still allow facilitation of learning when there is decreased in-person gatherings [1,2]. Online learning can pose challenges that could impact the learning of students, including the level of technical skills of the students and faculty, institutional support, attitudes regarding online learning, and resources available to devote to the implementation of digital learning [9].

The American College of Surgeons has published guidance for ATLS coordinators and training sites on how to conduct ATLS courses during the COVID-19 pandemic [3]. In addition to guidance on how to conduct socially distanced didactic lectures and skill stations, the American College of Surgeons explained the utility of mATLS during the pandemic [3]. The College stated that mATLS can be offered to ATLS students up to six months before their registered ATLS course. Due to the complications of COVID-19 and its effects on centers being able to host in-person ATLS courses, the College proposed that mATLS be used in order to allow residents to start their trauma rotations without having to complete the in-person activities of the ATLS course [3].

## Materials and methods

Design and setting

All of the ATLS courses were delivered at the USF Health Center for Advanced Medical Learning and Simulation (CAMLS). The content of ATLS remained consistent throughout the study. All courses had the same course director and coordinators. This study had an IRB waiver as the results of this study will be used for improvement of the ATLS course (IRB#: Pro00019597).

Baseline (Control) Course

An ATLS course completed in July 2019 included the traditional activities, that is, didactic lectures, skill stations, multiple-choice post-test, and procedural skills assessment. The post-test and skills assessment scores provided baseline data with which to compare the altered formats described next.

mATLS Courses

Two courses were delivered in September 2020. Both employed the mATLS, which is a program containing 13 online modules that cover the same material traditionally covered in the didactic lectures. Learners completed the mATLS before arriving at USF Health CAMLS [4]. Because mATLS contains the same material as the traditional ATLS didactic lectures in the baseline condition, the mATLS students only completed the skill stations, post-test, and the skills assessment at USF Health CAMLS (see Appendix A for detailed agenda).

Augmented Courses

In contrast, the three courses were delivered during the period October through December 2020. These were registered as traditional face-to-face ATLS courses. However, these courses differed from the baseline course in that students completed mATLS before attending the in-person course (see Appendix B for the agenda for the Baseline and Augmented courses).

Summary of Differences in Condition

Thus, in the pre-Covid baseline course, the students were exposed to the didactic material only in face-to-face lecture, and in the Covid mATLS courses, the students were exposed to the didactic material only by online programs. In the augmented courses, students were exposed to the didactic material twice, both electronically and then face-to-face. Otherwise, the courses were the same except being delivered at different months (all contained the same skill stations, post-test and skill assessment).

Instruments and data collection

The study cohort was PGY-1 surgical and emergency interns in six ATLS courses delivered at CAMLS from July 2019 to December 2020. 

The July 2019 course had 47 PGY-1 learners and a total of 48 learners (1 non-PGY-1 learner), the September 2020 courses had 45 PGY-1 learners and a total of 46 learners (1 non-PGY-1 learner), and the October, November, and December 2020 courses had 16 PGY-1 learners and a total of 46 learners (30 non-PGY-1 learners).

To determine if the learning of the students could have been affected by the delivery of their ATLS course, the post-test scores were compared between the three ATLS course delivery methods employed at USF Health CAMLS: pre-COVID traditional course (baseline control), mATLS course, and the augmented course. The post-tests, which are composed of 40 multiple-choice questions, were administrated to all learners online at the site of USF Health CAMLS with learners being proctored by the Educational Coordinator. Successful posttest scores must be greater than or equal to 75% (raw score of 30).

 After completion of the post-test, the learners completed a feedback survey where they rated aspects of the ATLS course and suggested changes for future ATLS courses. These learner feedback surveys were used to assess the subjective views that students had of the course and to determine student satisfaction with the course delivery.

In addition to evaluating the students, interviews were conducted with the Educational Coordinator for the ATLS course at USF Health CAMLS, the Course Coordinator of the ATLS course at USF Health CAMLS, and the Course Director at USF Health CAMLS. All of these individuals were present for the ATLS courses studied. Each person was asked six standard interview questions regarding the ATLS courses:

1. What were the main challenges in planning the ATLS courses in light of COVID-19? How did you address those challenges?

2. What differed in the planning and implementation of the ATLS courses during the COVID-19 pandemic?

3. Were there any technical difficulties during the ATLS courses? If so, how were they addressed?

4. Was there any feedback that stood out to you from the participants about the three sets of ATLS courses (June 2019, September 2020, and October/November/December 2020)?

5. Was there any feedback that stood out to you from faculty about the three sets of ATLS courses (June 2019, September 2020, and October/November/December 2020)?

6. Reflecting back on the three sets of ATLS courses (June 2019, September 2020, and October/November/December 2020), is there anything that you would change in regard to planning and conducting the courses?

## Results

Post-test results

To effectively compare the three methods of course delivery, only data from learners in PGY-1 are reported, although data from more advanced learners were also collected. A statistical summary of data from all learners is available by contacting the corresponding author.

**Figure 1:**
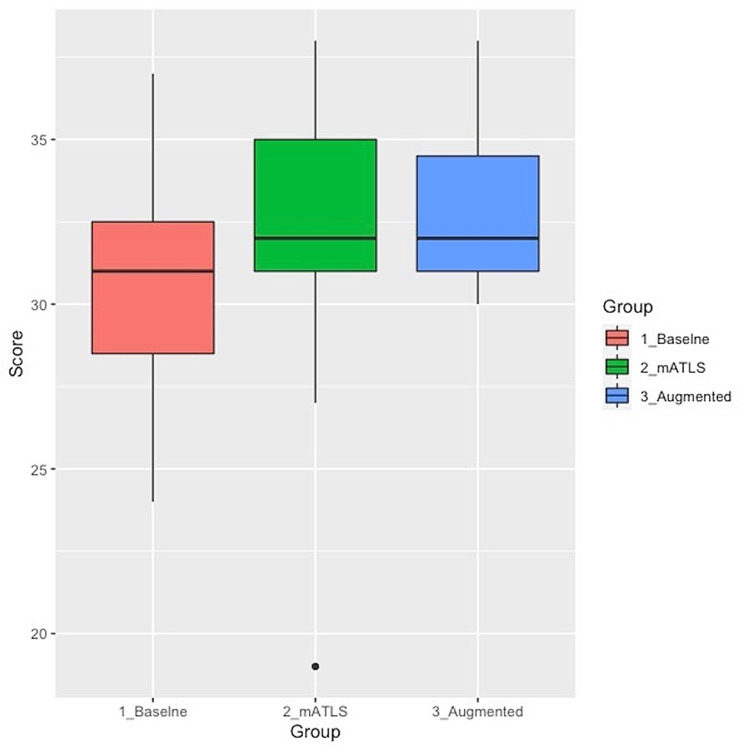
Post-test scores for just the Post Graduate Year One (PGY-1) learners in the baseline, mATLS, and augmented ATLS courses ATLS: Advanced Trauma Life Support, mATLS: mobile ATLS

The mean score, which is out of 40, among the PGY-1 learners (n=47) was 30.84 for the baseline control group. In comparison, the PGY-1 learners in the Covid mATLS group (n=45) had an average score of 32.42 and the PGY-1 learners in the augmented group (n=16) had an average score of 32.94. Figure 1 displays the distribution of the post-test scores among the three groups. Analysis of variance showed a significant difference among the groups [F(2,105) = 4.03, p < .05]. According to Tukey post-hoc tests, only the mATLS group was significantly different compared to the baseline group with a (p = 0.049). The augmented group fell short of being significantly different compared to the baseline group with a p-value score of 0.063 (but recall that the augmented group has a smaller sample size than the other groups). The Covid mATLS and augmented groups’ mean scores were not different from each other (p = 0.841). The pass rate (raw scores 30 or better) for all classes combined was 81%. Passing rates for the groups were Baseline = 68% (n=47), mATLS = 89% (n=45) and Augmented = 100% (n=16). 

Learner evaluation results

The evaluation surveys that the learners completed after the post-test had five questions. The survey asked learners for their comments and suggestions, what two things they are going to change in their practice as a result of the ATLS course, what barriers they think they will face while trying to implement those changes, what suggestions they have for future topics to be covered in future ATLS courses, and a space for any additional comments.

For the control course, there were 21 respondents to the survey out of the 48 learners. Twenty of the completed surveys responded with no suggested changes (e.g., “none” or left blank) to the survey question “Do you have suggestions for future topics to support and/or expand on what you have learned at this activity?” The most common response to the comments and suggestion section of the survey was the desire for more specific examples applicable to the post-test to be discussed and reviewed during the course with three mentions. In terms of “Describe the barriers you anticipate when implementing the above changes,” the most common responses were lack of experience with 3 mentions and the ability to recall and information during a trauma case with three mentions (e.g. “Remembering these in a stressful situation”).

For the Covid mATLS courses, there were 21 respondents to the survey out of the 46 learners. Twelve respondents did not make any suggested changes sections for the question “Do you have suggestions for future topics to support and/or expand on what you have learned at this activity?” For the barriers to implementation, there were four mentions of lack of experience and two mentions of decreased ability to practice due to the number of people present during a trauma case.

For the augmented courses, the comments were mostly positive responses with 10 mentions of the course instruction being excellent (e.g. “amazing course”). The most common response to the survey question of “Do you have suggestions for future topics to support and/or expand on what you have learned at this activity?” was no changes with 33 mentions (e.g. “nothing to change”). For barriers to implementing the lessons from the ATLS course, the most common response was lack of experience or exposure with 10 mentions and seven mentions of lack of equipment or institutional opposition (e.g. “Institutional barriers or possible pushback from attending preference”).

Interview results

The main challenge that the ATLS Course Director, ATLS Course Coordinator and the Educational Coordinator discussed was maintaining social distancing throughout the duration of the course while having the interactive sessions. The ATLS Course Coordinator also addressed how more staff was required in order to ensure that the proper health screenings and cleaning procedures were being adhered to during the course.

Both the ATLS Course Director and the ATLS Course Coordinator stated the planning and implementation of the 2020 ATLS courses differed from previous courses in that additional time had to be allotted to allow for proper disinfection of the skill stations and for proper socially distanced movement of the learners between stations and sessions. The Educational Coordinator said that the plans were able to be adhered to during the course with only moments of learners not properly socially distancing or getting lost. The ATLS Course Director, ATLS Course Coordinator, and the Educational Coordinator all stated that there were no major technical difficulties. The simulation team was able to keep the stations moving and there was on-site IT staff in case there were any technical issues.

The ATLS Course Director, ATLS Course Coordinator and the Educational Coordinator said that learners did complain about the amount and length of wait times through the ATLS courses due to cleaning or testing. Learners overall reported that they felt like they received a good education with the different ATLS courses.

The ATLS Course Director and the Educational Coordinator explained that faculty were pleased with measures like station guides being provided to help support them at the skill stations. The ATLS Course Coordinator said that faculty were unsure of what to do during the decontamination times in between stations with some thinking that it was break.

With regard to changes for future ATLS courses, the ATLS Course Director and the Educational Coordinator suggested more support whether in the form of more ancillary staff or in-house radios between simulation staff and the coordinators. The ATLS Course Coordinator suggested that a meeting could be done before the ATLS course in order to ensure that all of the faculty know the schedule and expectations.

Overall, the ATLS Course Director, ATLS Course Coordinator, and the Educational Coordinator said that the ATLS courses were still able to effectively instruct learners even with differences in the schedules between the courses. Detailed data from the interviews are available by contacting the corresponding author.

## Discussion

The COVID-19 pandemic increased the need for distanced learning generally, and ATLS courses specifically. Conventional ATLS courses are two days long, costing providers time and money to travel in order to attend and participate in the course, especially for healthcare providers from rural communities. Having a digital platform like mATLS may shortening the length of in-person attendance and thus increase accessibility for healthcare providers.

The results of this study demonstrate that mATLS was able to be used successfully as a replacement for in-person lectures for the ATLS courses at USF CAMLS. Scores on the post-test for learners exposed to the mATLS only were significantly greater on average than those who were exposed to the same material face-to-face only. Mean score for the augmented group, in which delivery included both mATLS and face-to-face, was similar to that for the mATLS group. Although the differences in the post-test means are not large, the greater passing rates in classes including mATLS compared to the baseline class suggests a practical advantage to including mATLS . Overall, the results support the inference that the mATLS modules provided instruction about factual information (declarative knowledge) at least as well as did face-to-face instruction at USF Health CAMLS.

The survey results from the learners and the interviews of the coordinators for the three sets of ATLS courses did not reveal any major differences between the three delivery methods of ATLS. A difference in the survey results between the ATLS course from 2019 and the ATLS courses from 2020 was that there was an increase in learners responding to the question “Describe the barriers you anticipate when implementing the above changes,” saying that lack of experience or exposure to trauma care were potential barriers in implementing what they have learned from ATLS. During the COVID-19 pandemic residents may have had decreased exposure to a diversity of cases, so this topic may serve as an area of future research in seeing if the COVID-19 pandemic will affect the performance and confidence of residents.1[BM1] 

Although the results were promising for utilization of mATLS in ATLS courses, a limitation to generalizability of this study is the small number of PGY-1 participants in a single instructional site. Broader generalizability regarding the comparative efficacy of mATLS and face-to-face instruction could be enhanced with a multi-site study that has a greater diversity of healthcare professionals, including physician assistants and nurse practitioners. A second limitation of the study is that three of the authors of the paper were also interviewed for their perspectives, thus providing qualitative data for the study and a possible source of bias in the results. However, the authors’ data are consistent with those of the students in the courses. Further, their data had no impact on the quantitative analyses of the post-test data. Therefore, we included the interview data for sake of completeness.

## Conclusions

The COVID pandemic has impacted the delivery of medical education throughout the world. Novel approaches to traditional face-to-face courses, including various online formats, have resulted in modifications of instruction to achieve knowledge and skill acquisition. mATLS offers an online platform for didactic (knowledge) instruction, which can be followed by face-to-face skills rotation and evaluation, thus reducing face-to-face interaction. The well-established face-to-face delivery at a premier simulation center that had delivered ATLS for over eight years proved an ideal environment to evaluate the new modality.

This preliminary study analyzed three delivery models for ATLS courses. Delivery by mATLS may replace in-person delivery as students that received the mATLS delivery showed scores on the post-test that were as high or higher than those who received only face-to-face instruction. According to educators’ and learners’ comments, the main problems associated with ATLS instruction during Covid were difficulties in maintaining social distance, the extra time needed to clean hands-on stations during training and subsequent limits to resident exposure to real patients.

## Appendices

Appendix A: Agenda for the mATLS courses.

Day One

6:30-7:00 AM Participant Registration and Breakfast

7:00-7:35 AM Module Review Question and Answers with ATLS Course Director

7:35-8:00 AM Initial Assessment Introduction and Video with Question and Answer

8:00-8:15 AM Move to the Skill Stations

8:15 AM-10:50 AM Airway and Breathing Skill Stations

10:50 AM-11:20 PM Break while the Stations are Cleaned and Changed for the Next Skill Stations

11:25 AM-1:30 PM Circulation 1 and Circulation 2 Skill Stations

1:30-2:30 PM Lunch with Discussion of the Triage Scenarios

Day Two

6:30-7:00 AM Participant Registration and Breakfast

7:30-8:00 AM Question and Answer Session

8:00-8:10 AM Move to the Skill Stations

8:10-8:55 AM Team Initial Assessment with Secondary Assessment Skill Station

8:55-9:35 AM Break while the Stations are Cleaned and Changed for the Next Skill Stations

9:40-10:25 AM Disability Skill Station

10:30 AM-11:50 AM

Group 1: Practice for the Skills Assessment and Complete the Skills Assessment

Group 2: Complete the Written Test

12:00-12:20 PM Lunch

12:20-1:40 PM

Group 1: Complete the Written Test

Group 2: Practice for the Skills Assessment and Complete the Skills Assessment

Appendix B: Agenda for the Baseline and Augmented ATLS courses.

Day One

7:00-7:30 AM Participant Registration and Breakfast

7:30-7:45 AM Introduction and Course Overview

7:45-8:30 AM Initial Assessment Introduction and Video

8:30-9:00 AM Airway and Ventilatory Management Discussion

9:00-9:25 AM Thoracic Trauma Discussion

9:25-9:35 AM Break

9:35-10:05 AM Shock Discussion

10:05-10:35 AM Abdominal and Pelvic Trauma Discussion

10:35-11:05 AM Musculoskeletal Trauma Discussion

11:05 AM-12:15 PM Lunch and Discussion of the Pre-Test

12:15-12:30 PM Move to the Skill Stations

12:30-3:05 PM Airway and Breathing Skill Stations

3:05-3:35 PM Break while the Stations are Cleaned and Changed for the Next Skill Stations

3:40-5:15 PM Circulation 1 and Circulation 2 Skill Stations

Day Two

7:00-7:30 AM Spine Trauma Discussion

7:30-7:55 AM Pediatric Trauma Discussion

7:55-8:20 AM Geriatric Trauma

8:20-8:50 AM Thermal Discussion

 8:50-9:00 AM Break

9:00-9:30 AM Trauma in Pregnancy and Intimate Partner Violence Discussion

9:30-10:00 AM Move to the Skill Stations

10:00 AM-11:15 AM Team Initial Assessment with Secondary Assessment Skill Station

11:15-11:45 AM Lunch and Triage Scenarios

11:50 AM-12:35 PM Disability Skill Stations

12:40- 2:00 PM

Group 1: Practice for the Skills Assessment and Complete the Skills Assessment

Group 2: Complete the Written Test

2:05-3:25 PM

Group 1: Complete the Written Test

Group 2: Practice for the Skills Assessment and Complete the Skills Assessment
